# Heparinized Polyurethane Surface Via a One-Step Photografting Method

**DOI:** 10.3390/molecules24040758

**Published:** 2019-02-20

**Authors:** Zhangshuan Liu, Liming Fang, Guillaume Delaittre, Yu Ke, Gang Wu

**Affiliations:** 1 National Engineering Research Center for Tissue Restoration and Reconstruction, Guangzhou 510006, China; X_recon@163.com (Z.L.); lmfang@scut.edu.cn (L.F.); 2 School of Medicine, South China University of Technology, Guangzhou 510641, China; 3 School of Materials Science and Engineering, South China University of Technology, Guangzhou 510641, China; 4 Institute of Toxicology and Genetics (ITG), Karlsruhe Institute of Technology (KIT), 76344 Eggenstein-Leopoldshafen, Germany; guillaume.delaittre@kit.edu; 5 Institute for Polymer Chemistry and Chemical Technology (ITCP), Karlsruhe Institute of Technology (KIT), 76137 Karlsruhe, Germany; 6 Department of Biomedical Engineering, Jinan University, Guangzhou 510632, China

**Keywords:** catheter, polyurethane, photografting, heparinization, bioactivity

## Abstract

Traditional methods using coupling chemistry for surface grafting of heparin onto polyurethane (PU) are disadvantageous due to their generally low efficiency. In order to overcome this problem, a quick one-step photografting method is proposed here. Three heparin derivatives incorporating 0.21, 0.58, and 0.88 wt% pendant aryl azide groups were immobilized onto PU surfaces, leading to similar grafting densities of 1.07, 1.17, and 1.13 μg/cm^2^, respectively, yet with increasing densities of anchoring points. The most negatively charged surface and the maximum binding ability towards antithrombin III were found for the heparinized PU with the lowest amount of aryl azide/anchor sites. Furthermore, decreasing the density of anchoring points was found to inhibit platelet adhesion to a larger extent and to prolong plasma recalcification time, prothrombin time, thrombin time, and activated partial thromboplastin time to a larger extent. This was also found to enhance the bioactivity of immobilized heparin from 22.9% for raw heparin to 36.9%. This could be explained by the enhanced molecular mobility of immobilized heparin when it is more loosely anchored to the PU surface, as well as a higher surface charge.

## 1. Introduction

Venous catheters are typically placed into a large vein and used to administer medication or fluids, obtain blood tests, and measure central venous pressure [1]. After being placed in vivo for an extended period of time, a catheter faces the risk of blood clots, which is critical for cancer patients as they are particularly inclined to develop these [2]. Anti-clotting drugs such as heparin and fondaparinux decrease the incidence of blood clots. However, due to the difficulty in dosage control, these drugs may possibly cause bleeding, pain, low platelets level, and even thrombocytopenia [3,4]. To decrease the potential risks and adverse effects, methods including physical entrapment, as well as ionic and covalent surface binding of heparin or other substances, have been utilized to enhance catheter anticoagulation properties [5]. 

Compared to physical adsorption or entrapment, covalently heparinizing the surface of catheters provides a robust anticoagulation coating which can withstand long-term exposure in vivo [6]. Covalent surface immobilization of heparin reported in the literature generally entails multiple complex steps comprising several surface activation and coupling chemistry reactions [7], limiting the translation of this technology to a more industrial/commercial context. In addition, the compromised bioactivity and the difficulty of increasing the grating density are other problems which need to be overcome. 

It was reported that the bioactivity of affixed heparin decreased to 22.4% as compared to raw heparin, even the grafting density was increased up to 1.4 μg/cm^2^, due to the restrained surface mobility after immobilization [8]. Consequently, spacer arms have been employed as “bridges” between heparin and the substrate surface. Spacers offer the affixed heparin room for molecular spatial rotation, thereby exposing its bioactive anticoagulation sequences [9,10]. The increase of heparin grafting density could enhance anticoagulation, but since heparin is in general negatively charged in aqueous solution, once it is immobilized, it would repel extra heparin approaching the surface. Some other methods have also been developed to improve anticoagulant efficiency of immobilized heparin. For example, covalent binding of heparin together with laminin yielded a biocompatible and multifunctional surface with better anti-thrombosis [11]. 

It is expected that a better immobilization method could simultaneously meet the requirements for yielding an increase in heparin bioactivity and grafting density, as well as for industrial translation. Especially nowadays, heparinized surfaces are more and more widely applied to bind and release growth factors and for cell retention besides simple hemocompatibility enhancement [12,13,14,15]. An improved immobilization strategy is therefore worth exploring. 

Photografting is the covalent incorporation of functional moieties to a material matrix or onto a surface using a light-induced mechanism [16,17,18]. For example, Giol et al. have introduced gelatin at the surface of polyethylene terephthalate by using a photografting method to improve the endothelialization and anticoagulation potential for vascular applications [19]. This single-step, versatile, and spatially controlled protocol (μm to a few hundreds of nm) with very good reproducibility is important for surface functionalization and has been widely used in industry [20]. However, to the best of our knowledge, very few efforts have been dedicated to using this technology for immobilizing heparin onto biomaterial surfaces [21,22]. 

In this work, heparin derivatives modified with pendant photosensitive groups were synthesized and were grafted onto polymer surfaces through a C-H insertion mechanism after light irradiation. Surface characterization after heparin immobilization was assessed by water contact angle and bovine serum albumin (BSA) adsorption. The amount of immobilized heparin was quantified by toluidine blue assay. The binding capability of antithrombin III to heparinized polyurethane (PU) film was characterized by quartz crystal microbalance (QCM), and the anticoagulant properties were evaluated by platelet adhesion, prothrombin time (PT), thrombin time (TT), and activated partial thromboplastin time (APTT). 

In analogy to the immobilization strategy employing spacer arms, considering that restriction on molecular mobility impacts the exposure of bioactive sequences, the extent of the immobilization sites of heparin was assessed against anticoagulation properties. Three heparin derivatives with various amounts of pendant groups were expected to be grafted onto PU with differing numbers of grafting sites, leading to disparate surface mobility. The impact of the variation of anchoring points on heparin bioactivity was investigated.

## 2. Results

### 2.1. Heparin Grafting onto PU Surface

**Figure 1:**
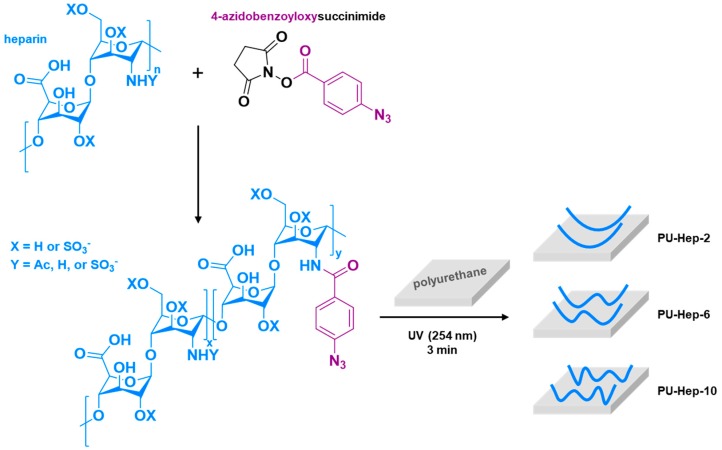
Synthetic route for the photoreactive heparin derivatives and schematic illustration for their phototriggered immobilization onto polyurethane (PU) films.

Heparin derivatives containing various amounts of pendant azido-benzoic groups were synthesized by reacting heparin with *N*-(4-azidobenzoyloxy)succinimide for different periods of time (see Figure S1). Upon UV irradiation, heparin-bound aryl azides are converted to highly reactive nitrenes which can easily insert into C–H bonds and yield amines [23]. As illustrated in Figure 1, the heparin derivatives were then immobilized covalently on the PU surface following this mechanism. Heparin derivatives possessing more pendant azide groups are expected to be immobilized through more anchoring sites, as displayed in Figure 1.

### 2.2. Characterization of Heparin on Surface

**Figure 2:**
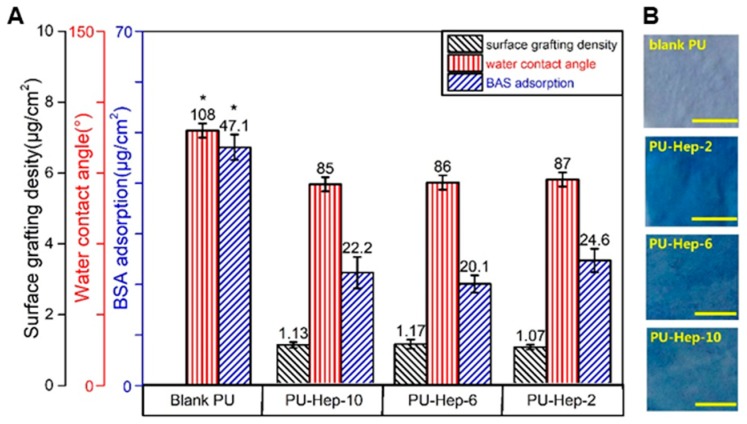
(**A**) Heparin grafting density, water contact angle, and amount of adsorbed bovine serum albumin (BSA). (**B**) Photographs of PU and heparinized PU films after toluidine blue staining. The scale bars represent 500 μm.

After hydrophilic heparin immobilization, the average water contact angle decreased from 108° for the pure PU film to 85–87° for all heparinized films (Figure 2). The amount of BSA adsorbed onto the heparinized PU films decreased to 20–25 μg/cm^2^, significantly lower than 47.1 μg/cm^2^ for the pure PU film. Both water contact angle and BSA adsorption showed significant differences compared to the pure PU film (*p* < 0.05), but no significant difference among heparinized films. After staining with toluidine blue, a homogeneous blue color could be visualized on all heparinized PU films, while the color of the pure PU film remained unchanged. These results suggest that heparin was homogenously grafted onto the PU film surface. The heparin amount on these films can also be quantified by the toluidine blue assay, revealing average grafting densities of 1.07, 1.17, and 1.13 μg/cm^2^ for PU-Hep-2, PU-Hep-6, and PU-Hep-10, respectively (Figure 2), with no significant differences among them.

**Figure 3:**
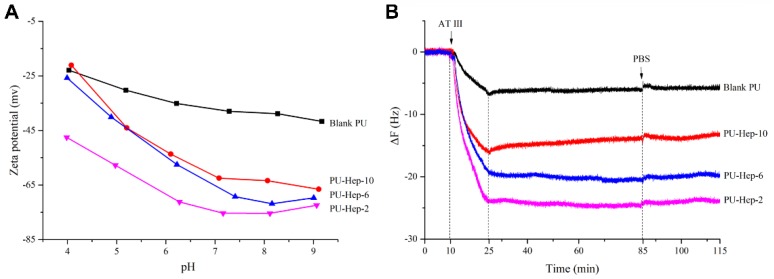
(**A**) Zeta potential at various pHs and (**B**) quartz crystal microbalance (QCM) measurements for PU and heparinized PU films.

The influence of the amount of anchoring points on the molecular mobility of immobilized heparin was investigated by surface zeta potential and QCM tests. Surface zeta potential gives information on the surface charge. The zeta potential of both PU-Hep-10 and PU films decreased when going from pH 4 to pH 9 (Figure 3A). In contrast, the zeta potential of PU-Hep-6 and PU-Hep-2 showed a minimum value around pH 7 and 8. It was also witnessed that the surface zeta potential of PU-Hep-2 was the smallest of all surfaces, irrespective of the pH value. These results show that the exposure of negatively charged groups by immobilized heparin to the surface could be enhanced in the case of a looser attachment, suggesting that the heparin with less anchor points may extend further away from the surface and present higher molecular mobility.

The molecular mobility was further probed with QCM measurements using antithrombin Ⅲ (AT-III) conjugation to the heparinized surface (Figure 3B). The binding of antithrombin III to heparin is mediated by a well-defined unique heparin pentasaccharide sequence that induces a conformational change in the antithrombin, enhancing its ability to inhibit factor Xa and related serine proteases in coagulation cascade [24]. In QCM measurements, frequency declines if the weight on the surface increases [25]. As seen in Figure 3B, the frequency was stable during PBS rinsing in the first 10 minutes. After AT-III was introduced to the chamber, a strong reduction in frequency took place, indicating the adsorption/conjugation of AT-III to the sensor surface. The frequency did not decline any further after the injection of AT-III was stopped (at 25 min), suggesting the quick conjugation of AT-III to the surface. Furthermore, no increase of frequency was found during thorough PBS rinsing (from 85 min on), suggesting no desorption of AT-III from the surface and therefore indicating firm and irreversible binding. A similar adsorption/behavior pattern was also noticed for AT-III on the blank PU surface, possibly due to unspecific adsorption. 

The scope of frequency reduction was linked to the weight of AT-III adsorbed onto the sensor surface. As that displayed in Figure 3B, the amount of AT-III increased with decreasing amount of anchoring points in heparin derivatives. Since grafting density was similar for the three heparinized surfaces, the amount of AT-III on surface should be directly linked to the number of bioactive sequences exposed. The results point to more bioactive sequences exposed by the immobilized heparin whose molecular mobility was less restricted, i.e., with less anchor sites.

### 2.3. Hemocompatibility Evaluation

A comprehensive hemocompatibility evaluation was conducted with several methods, including hemolysis assay, plasma recalcification time (PRT), platelet adhesion, prothrombin time (PT), and activated partial thromboplastin time (APTT), as well as thrombin time (TT) tests. 

**Figure 4:**
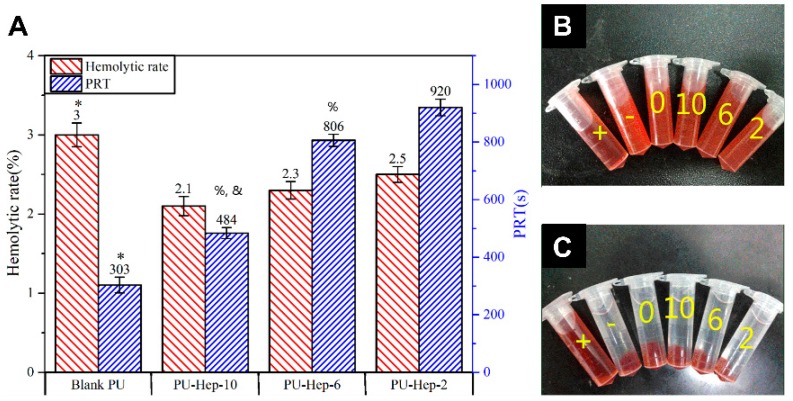
(**A**) Hemolytic rate and plasma recalcification time (PRT) of PU and heparinized PU films. Samples marked with (*) are significantly different from PU film with immobilized heparin. Samples marked with (%) are significantly different from the PU-Hep-2 group (*p* < 0.05). Samples marked with (&) are significantly different from the PU-Hep-6 and PU-Hep-2 groups (*p* < 0.05). (**B**,**C**) Photographs of blood/saline mixture before (B) and after (C) centrifugation. Labels 0, 2, 6, 10, - and + represent the blank PU, PU-Hep-2, PU-Hep-6, PU-Hep-10 films, negative and positive controls, respectively.

The hemolysis of the blank PU, PU-Hep-2, PU-Hep-6, and PU-Hep-10 films were 3.0, 2.5, 2.3, and 2.1% (Figure 4A), respectively. It decreased after heparin was grafted onto the bare PU surface and the hemolysis of all heparinized films was lower than 5%, i.e., the clinical diagnosis benchmark for blood-compatible biomaterials. Figure 4B,C illustrate the supernatant color before and after centrifugation in the hemolysis assay. Due to the disruption of the erythrocyte membrane in the positive control group, hemoglobin released into the medium induced a solution color change to red. On the contrary, no color could be visualized for the negative control and the groups with various PU films since intact erythrocytes were pelleted at the bottom of the tube after centrifugation.

Plasma recalcification time (PRT) is an assay evaluating the intrinsic pathway of coagulation triggered by implanted biomaterial. A longer PRT indicates better anticoagulation performance. Compared to the blank PU showing a PRT of 303 s, PRT significantly increased after heparin photografting. PRT of 484, 806, and 920 s were determined for PU-Hep-10, PU-Hep-6, and PU-Hep-2 films, respectively (Figure 4A). The longest PRT of PU-Hep-2 indicated it had the best trigger resistance against blood coagulation cascades. 

**Figure 5:**
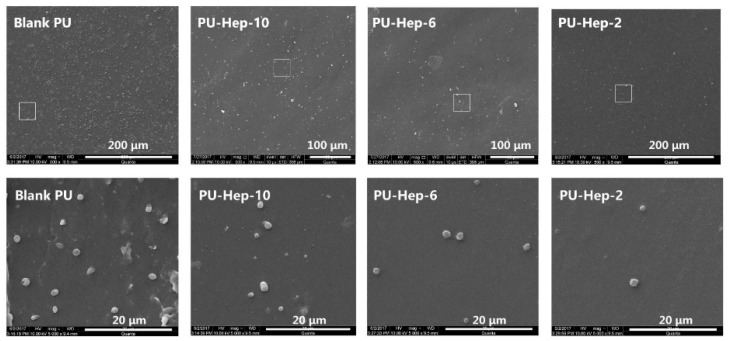
SEM images of platelet-rich plasma (PRP) morphology being contacted with PU and heparinized PU films.

The platelet adhesion extent is positively relevant to thrombus formation and is used to evaluate biomaterials blood compatibility. In Figure 5, it is observed that the amount of surface-adhered platelets decreased after heparin photografting. The minimum amount of adhered platelets was found on PU-Hep-2. It is possibly due to the prevention of protein adsorption from plasma by the most negatively charged PU-Hep-2 surface (Figure 3A).

The results of the anticoagulant activity evaluation using PT, APTT, and TT tests are shown in Table 1. Compared to the control, a normal pooled plasma, the test values for the blank PU film varied only marginally, suggesting the blank PU had minor impact on the normal coagulation cascade pathway. After heparin immobilization, the test values of PT, TT, and APTT were all significantly prolonged for the three heparin immobilized surfaces. For all three tests, times were prolonged to the largest extent for the PU-Hep-2 group. The highest bioactivity (36.9%) was also measured from this group, suggesting the immobilized heparin on the surface presented its bioactive sequences better than it did on PU-Hep-6 and PU-Hep-10. 

### 2.4. Cell Assays

**Figure 6:**
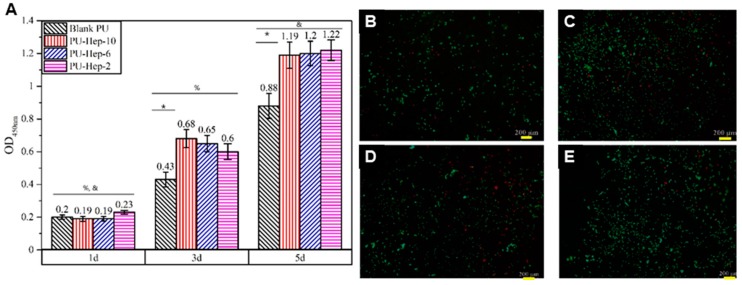
Cell proliferation (**A**) and live/dead cell staining on blank PU film (**B**) and heparinized PU films: PU-Hep-10 (**C**), PU-Hep-6 (**D**) and PU-Hep-2 (**E**). Within a group, sample marked with (*) are significantly different from heparinized PU films at the same time point (*p* < 0.05). Between groups, the group noted with (%) are significantly different (*p* < 0.05) compared to day 5, and with (&) are significantly different (*p* < 0.05) compared to day 3. (scale bars = 200 µm).

Cell proliferation and viability were evaluated using the CCK-8 method and live/dead cell staining. The increasing optical density (OD) with culture time indicated cell proliferation on all PU films (Figure 6A). The enhanced OD value suggested cell proliferation was greater on the heparinized PU films than on the blank PU, yet no significant difference was observed among the three heparinized PU films. Staining images of the live/dead cells on all kinds of PU films at day 3 (Figure 6B–E) showed more live cells (dotted green) on the heparinized PU films, corroborating the trends observed in OD. However, a small portion of dead cells (in red) on the heparinized PU films could still be visualized. Compared to that on blank PU film, the dead cell ratio was smaller, suggesting cell viability was greater on the heparinized PU films. 

## 3. Discussion 

For clinical applications, heparinizing a catheter surface by covalent bonding provides long-lasting anti-thrombogenicity. Heparin has previously been attached to inert catheter surfaces via carbodiimide coupling or other methods after surface activation of carboxyl or amine groups [11]. Our study shows that with this one-step UV irradiation, heparin derivatives can be successfully grafted onto PU surfaces. Compared to traditional carbodiimide coupling methods, UV irradiation is faster and offers higher precision. These attributes are particularly interesting because catheter extrusion for massive industrial manufacturing is generally a fast continuous process, where a rapid photografting step could be ideally combined. 

In addition to this, another potential advantage of the phototriggered “grafting to” method through C–H insertion over the traditional carbodiimide coupling method is the reduced amount of foreign chemical moieties introduced to the applied surface, since carboxyl or amine groups could potentially induce unexpected reactions, such as the activation of plasma proteins and acceleration of platelets adhesion [8]. 

To achieve better anti-thrombogenicity, heparin should be grafted at a high density onto the catheter surface. However, due to the intrinsic electrostatic repulsion between negatively charged heparin molecules, the first grafted molecules preclude further heparin molecules from approaching the surface. In our experiments, it was also found that higher concentrations are required for heparin incorporating less aryl azide groups to obtain similar surface grafting density, probably because this heparin derivative retains more negative charges. UV irradiation can also be conducted on totally dry coatings in which the photosensitive heparin derivatives are adsorbed, effectively avoiding electrostatic repulsion in the solution. This is another merit of this technique over the traditional coupling method conducted in solution, although we do not demonstrate here how much heparin could be grafted to the surface using this method. 

The function of heparin tightly binding to a surface could be compromised if its biological active sequences responsible for anticoagulation are not exposed correctly [26], even if only small portions composed of a pentasaccharide sequence and another 18-saccharide unit are responsible for blocking the activation of coagulation factor Xa and thrombin [27,28]. To improve this, spacer arms have earlier been introduced between heparin and the surface to increase heparin mobility as well as bioactivity. For example, Park et al. investigated the impact of several hydrophilic spacers on heparin bioactivity and found that polyethylene oxide spacers with a molecular weight of 4000 showed the best bioactivity (19% in comparison to raw heparin) [29].

Here, although heparin was directly grafted onto the biomaterial surface without spacer, a strategy for enhancing the molecular mobility and the capability of heparin to expose its bioactive sequences was achieved by considering the extent of anchoring points. Correspondingly, the heparin derivative bearing the lowest amount of pendant photoreactive groups, that is, which possesses the lowest number of potential anchors, presented the best anticoagulant property. The bioactivity of the immobilized heparin increased from 22.9 to 36.9%, with an almost constant grafting density of 1.1 μg/cm^2^ when decreasing the concentration of pendant photosensitive aryl azide groups from 8.8 to 2.1 wt‰. Compared to the bioactivity of 22.4% for heparin grafted by traditional methods, even with a grafting density of 1.4 μg/cm^2^ [8], this is a significant improvement in the area of heparinized surfaces, obtained through increasing molecular mobility.

## 4. Experimental Section

### 4.1. Materials

Three heparin derivatives containing various weight percentages of pendant azidobenzoic photosensitive groups (2.1, 5.8, and 8.8‰) were synthesized (see Figure S1) and termed Hep-2, Hep-6, and Hep-10, respectively. PU (Lubrizon, Estane®2103-70A) was provided by the Healthline Co. Ltd (Foshan, China). BSA was bought from Qiyun biotechnology Co. Ltd (Guangzhou, China). Phosphate buffer saline (PBS) was obtained from Gibco (Shanghai, China). Antithrombin III (HCAT III-0120, Haematologic Technologies) was purchased from Pumai Biotechnology (Shanghai, China). All the other chemicals (analytical grade) were obtained from Sinopharm Chemical Reagent (Shanghai, China) and used without further purification. 

Dulbecco’s Modified Eagle Medium (DMEM/F12) and fetal bovine serum (FBS) were from Gibco (Shanghai, China). The cell-counting kit-8 (CCK-8) and Live/Dead cell staining kit were bought from Donjindo (Shanghai, China). The micro BCA™ protein assay reagent kit (51254-1KT) was obtained from Sigma-Aldrich (Shanghai, China). The RecombiPlasTin 2G kit (Instrumentation Laboratory, Barcelona, Spain), Thrombin Time kit (Instrumentation Laboratory, Barcelona, Spain) and SynthASil kit (Instrumentation Laboratory, Barcelona, Spain) for blood compatibility tests were purchased from Qiyun Biotechnology Co. Ltd (Guangzhou, China). The ATCD5 cell line was purchased from Jiniou Co. Ltd (Guangzhou, China). 

Healthy human fresh blood from a volunteer was collected in anticoagulant tubes (2.7 mL, BD VACUTAINER, Shanghai ThreeBio Technology, Shanghai, China) and centrifuged at 1000 rpm for 15 min to obtain platelet-rich plasma (PRP) or at 4000 rpm for 15 min to obtain platelet-poor plasma (PPP), respectively [30].

### 4.2. Heparin Surface Grafting

PU films prepared using solvent casting and evaporating methods were immersed in three plastic molds containing 20 mL PBS solution at concentrations of 40, 15, and 10 mg/mL for Hep-2, Hep-6, and Hep-10, respectively. The molds were transferred to a dark desiccator allowing water evaporation and then irradiated under a UV lamp (8 W, 254 nm) for 3 min at a distance of 30 cm away from the lamp. Ultrasonic cleaning was applied to the resulted films in a 0.05% NaOH solution, followed by deionized water three times. The dry heparinized PU films were cut into 1 cm × 1 cm squares for tests.

### 4.3. Characterization

The water contact angle on film was measured with a Kruss DSA25 instrument (Hamburg, Germany) via the sessile-drop method at 25 °C. The surface zeta potential of the films was measured at 25.0 ± 0.5 °C in a pH range from 4.0–9.0 using a SurPASS device (Anton Paar, Graz, Austria). BSA adsorbed on film was determined by a Micro BCA™ protein assay reagent kit as the following steps. The film (1 cm × 1 cm) was immersed in 2 mL BSA/PBS solution (1 mg/mL) for 2 h at 37 °C, rinsed with PBS, and washed in an ultrasonication bath at 37 °C in 2 mL aqueous SDS (2 wt.%, 0.05 M NaOH) for 30 min. The amount of adsorbed BSA was calculated by subtracting the eluted BSA in SDS solution which was quantified using the BCA method from the total BSA in the original PBS solution [31].

The quantity of surface-immobilized heparin was determined using the toluidine blue method by measuring absorbance at 631 nm with a UV-vis spectroscope (TU-1901, Persee, Beijing, China) [32]. A calibration curve was initially prepared. Briefly, 1 mL heparin solutions with known concentrations (1–5 μg/mL in 0.2% NaCl) were added to 5 mL 0.01 N HCl homogenous solution containing 25 mg toluidine blue. Following that, the mixed solution was added to 3 mL *n*-hexane and vortexed for 30 s to extract the heparin/toluidine blue complex. The plot of the absorbance regarding the known heparin concentration was then obtained by immediate measurement of residual toluidine blue in the aqueous layer. The amount of heparin on films was quantified according to the plotted calibration curve using the same procedure. A bare PU film was used as control.

QCM measurements were performed on a Q-Sense E1 device (Omega Auto, Stockholm, Sweden) using gold-coated AT-cut quartz crystal electrode sensor (natural frequency 5 MHz), which was cleared and dried as previously reported [33]. A homogeneous PU layer on the sensor was prepared by spin-coating a 0.5 wt.% PU/*N*,*N*-dimethylformamide solution using an CHY-EZ4 spin coater (Chenyi, China) at 2000 rpm for 50 s, followed by drying for 2 h under vacuum. The surface of PU-coated sensors was then heparinized as described above using photoirradiation. The resulting sensor placed in the measurement chamber was first rinsed with PBS (pH 7.4) at a flow rate of 150 μL/min and 37 °C until the signal was stable. Then, an antithrombin III (AT-III) PBS solution (10 μg/mL) was introduced to the chamber during 15 min at the same flow rate, followed by a resting period of 60 min allowing AT-III conjugation. Finally, the sensor was flushed with PBS at a flow rate of 150 μL/min to remove the unconjugated AT-Ⅲ. The frequency change during the rinsing was recorded. 

### 4.4. Blood Compatibility Tests

Platelet adhesion on the PU films was performed as follows [34]. The film samples (1 cm × 1 cm) were incubated with 0.5 mL of equilibrated fresh PRP at 37 °C for 120 min. They were rinsed by PBS three times, followed by immersion in a 2.5 wt% glutaraldehyde solution in PBS at 4 °C for 4 h. The films were washed with PBS, air dried, gradient dehydrated in a series of alcohol-PBS solutions (30, 50, 70, 80, 90, 95, and 100%) and subsequently in isoamyl acetate-alcohol solutions (30, 50, 70, 80, 90, 95, and 100%), and freeze-dried with a lyophilizer (FD-1C-50, Boyikang, Beijing, China). The morphology of the platelets on the films was then observed using a Q25 microscope (FEI, Hillsboro, America) after gold sputtering. 

Hemolytic evaluation was assayed by incubating PU films (10 mm × 25 mm) in 10 mL saline at 37 °C for 30 min followed by incubation with extra 0.2 ml blood in the saline for another 60 min [35]. The optical density (OD) of the supernatant after centrifugation at 2500 rpm for 10 min was measured with a Thermo3001 microplate reader (Thermo Scientific, Waltham, America) at the wavelength of 545 nm. The blood sample mixed with distilled water and saline were used as positive and negative controls. Hemolytic rate (HR) was calculated according to the equation: HR = (OD_s_ − OD^−^)/(OD^+^ − OD^−^), where OD_s_, OD^+^, and OD^−^ were denoted as the OD values of the sample group and positive and negative controls, respectively.

Plasma recalcification time (PRT) of PU films was assayed by immersion of the PU films (1 cm × 1 cm) in PBS at 37 °C for 1 h followed by adding 100 μL PPP and 100 μL of a 25 mM CaCl_2_ aqueous solution. Then, a stainless-steel hook coated with silicone was manually dipped in and took out from the mixture to record the clotting time when the fibrin formation on the hook was visible to the naked eye [5]. 

The blood anticoagulant activity of PU films conducted on an automatic blood coagulation analyzer STA-R (Diagnostica Stago, Paris, France) was tested using three techniques: Activated partial thromboplastin time (APTT), prothrombin time (PT), and thrombin time (TT). The PU films (0.5 cm × 0.5 cm) were incubated with 0.2 mL PPP in a transparent plastic tube at 37 °C for 30 min, and PT, TT, and APTT were measured using RecombiPlasTin 2G kit, Thrombin Time kit, and SynthASil kit, respectively [36]. The bioactivity of the immobilized heparin was calculated by comparing TT to the TT using pure heparin as a control. 

### 4.5. Cell Viability

The films (1 cm × 1 cm) were wetted in the culture medium at 37 °C for 3 h and placed into 24-well cell culture plates. ATDC5 were seeded onto the films at ~2.5 × 10^4^ cells/cm^2^, and blank PU film were used as a control. After 1, 3, or 5 days of culture, the fresh medium containing 10% CCK-8 solution was added into the wells, and the plate was incubated for 2 h. Then, 100 μL of the medium was transferred into a 96-well plate, and the absorbance at 450 nm was used to measure the viability of ATDC5. Live/Dead cell staining was used to detect the viability of ATDC5 cells at day 3 in four groups following the supplier instructions. The viable cells were stained with green fluorescent calcein while the dead cells were stained with red fluorescent ethidium homodimer II.

### 4.6. Statistical Methods

All statistical analyses were performed with the SPSS software. Statistical significance was determined by analysis of variance (ANOVA) followed by Fisher’s LSD with a significance level of *p* < 0.05. The results were displayed as mean ± SD, and all experiments were performed three times. 

## 5. Conclusions

Heparin derivative containing pendant aryl azide groups could be grafted onto PU films by UV irradiation. Three heparinized PU surfaces having similar heparin densities yet distinct densities of anchor points were achieved and characterized by water contact angle, BSA adsorption, and toluidine blue staining for quantification. They presented various zeta potentials and capabilities towards AT-III binding, as well as anti-coagulation properties. The number of pendant groups contained in these derivatives directly influenced the performance of surface-bound heparin, reflecting the importance of the density of anchor points on molecular mobility and access to bioactive sequences. It was found here that the immobilized heparin possessing the lowest amount of aryl azide exhibited the best anticoagulant performance, which is directly linked to the greatest molecular mobility. Via this strategy, the bioactivity of the immobilized heparin could be increased from 22.9 to 36.9%. Considering the advantages of this phototriggered “grafting to” method in terms of simplicity of implementation, fast occurrence, high surface grafting density, and enhanced heparin bioactivity, it could be a promising strategy applied to the continuous manufacture of catheters in the future.

## Figures and Tables

**Table 1:** Coagulant time (prothrombin time (PT), thrombin time (TT), activated partial thromboplastin time (APTT), and heparin bioactivity of PU and heparinized PU films.

Samples	PT (s)	TT (s)	APTT (s)	Bioactivity (%)
**Control**	12.4 ± 0.4	18.5 ± 0.5	35.7 ± 0.6	
**Blank PU**	12.9 ± 0.4 *	20.7 ± 2.3 *	40.8 ± 0.6 *	
**PU-Hep-10**	23.4 ± 1.1 ^%, &^	89 ± 2.9 ^%, &^	84.3 ± 1.3 ^%, &^	22.9 ^%, &^
**PU-Hep-6**	29.1 ± 0.9 ^%^	104.7 ± 3.2 ^%^	102.8 ± 2.2 ^%^	28.8 ^%^
**PU-Hep-2**	37.3 ± 1.5 ^&^	124.3 ± 3.5 ^&^	117.5 ± 4.4 ^&^	36.9 ^&^

(*) are significant differences (*p* < 0.05) compared to PU films tethering heparin; (^%^) are significant differences (*p* < 0.05) compared to PU-Hep-2; (^&^) are significant differences (*p* < 0.05) compared to PU-Hep-6.

## Supplementary Materials

The Supplementary Materials are available online. Preparation of the phenyl azido-heparin derivatives. Figure S1: FTIR and UV-vis spectra of azide-grafted heparin.
