# Supplementary material for: Heparinized Polyurethane Surface Via a One-Step Photografting Method
Source: Molecules. 2019 Feb 20;24(4):758. doi: 10.3390/molecules24040758 (PMC6412568; doi:10.3390/molecules24040758)
Supplement: Supplementary file 1 [file molecules-24-00758-s001.pdf]

Supplementary Materials

Heparinized Polyurethane Surface Via a One-Step Photografting Method

Zhangshuan Liu [1,2], Liming Fang [1,3], Guillaume Delaittre [4,5], Yu Ke [6,*] and Gang Wu [1,3,*]

[1] National Engineering Research Center for Tissue Restoration and Reconstruction, Guangzhou 510006, China
[2] School of Medicine, South China University of Technology, Guangzhou 510641, China;
[3] School of Materials Science and Engineering, South China University of Technology, Guangzhou 510641, China
[4] Institute of Toxicology and Genetics (ITG), Karlsruhe Institute of Technology (KIT), 76344 Eggenstein-Leopoldshafen, Germany
[5] Institute for Polymer Chemistry and Chemical Technology (ITCP), Karlsruhe Institute of Technology (KIT), 76137 Karlsruhe, Germany
[6] Department of Biomedical Engineering, Jinan University, Guangzhou, 510632 China

1. Preparation of phenyl azido-functionalized heparin

Reagents used for the synthesis. 1-(3-Dimethylaminopropyl)-3-ethylcarbodiimide hydrochloride (EDC), *N*-hydroxysuccinimide (NHS), anhydrous ethanol, and heparin sodium salt (205 USP units/mg) were purchased from Shanghai Macklin Biochemical Co. (Shanghai, China). Dimethyl sulfoxide (DMSO) was purchased from Tianjin Damao Chemical reagent Factory. 4-Azidobenzoic acid was obtained from Tokyo Chemical Industry Co., Ltd (Shanghai, China). Phosphate buffer saline (PBS) was obtained from Gibco (Shanghai, China). All other chemicals (analytical grade) obtained from Sinopharm Chemical Reagent Co. (Shanghai, China) were used without further purification. *N*-(4-azidobenzoyloxy)succinimide was synthesized according to a reported procedure [Matsuda T., Sugawara T. Photochemical protein fixation on polymer surfaces via derivatized phenylazido group. *Langmuir* **1995**, *11*, 2272].

The heparin derivatives incorporating various weight percentages of pendent photosensitive phenyl azido groups were synthesized as the following steps.

N-(4-azidobenzoyloxy)succinimide (0.1 g) was dissolved in DMSO (20 mL). Heparin sodium salt (1.0 g) dissolved in PBS (10 mL) was added to the *N*-(4-azidobenzoyloxy)succinimide solution under continuous stirring and was reacted for various periods of time (2, 6, and 10 h) at 4 °C. Then, the reaction mixture was dialyzed

against deionized water until *N*-(4-azidobenzoyloxy)succinimide could not be detected by monitoring UV absorbance at 267 nm from the dialysate solution. The resulting solution was lyophilized at – 36 °C to yield the final heparin derivatives. The obtained products were termed as Az-Hep-2, Az-Hep-6, and Az-Hep-10, according to the reaction time, respectively.

The derivatization of heparin was confirmed by IR and UV-Vis spectoscopies as displayed in Figure S1. FT-IR spectra (Figure S1A) of Hep-10, Hep-6, and Hep-2 showed the characteristic band at 2130 cm^{-1} ascribed to the azido group. Other characteristic absorption bands assigned to heparin, like 3470 cm^{-1} of O-H stretching vibration, 2948 cm^{-1} of C-H stretching vibration, 1716 cm^{-1} and 1640 cm^{-1} of C-H bonds bending vibration, 1146 cm^{-1} of S=O stretching vibration, were all present.

Figure S1B shows the UV-Vis spectra of derivatized heparin solution. The peak appearing at 267 nm is the characteristic absorption of phenyl azide. The amount of the pendant phenyl azido groups contained in derivatized heparin were obtained according to the calibration curve based on 4-azidobenzoic acid. The calculated weight percentages of incorporated 4-azidophenyl carboxyl groups were 0.21, 0.58, and 0.88 for Az-Hep-2, Az-Hep-6, and Az-Hep-10, respectively.

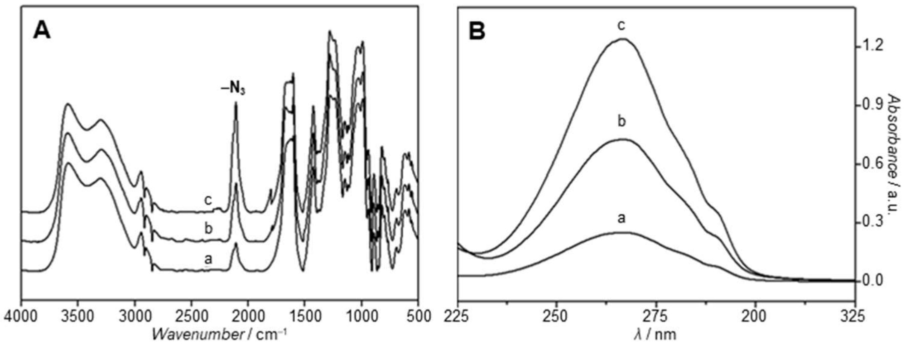

Figure S1. FTIR (A) and UV-Vis (B) spectra of phenyl azide-grafted heparin derivatives. Az-Hep-2, Az-Hep-6, and Az-Hep-10 were marked as a, b, and c, respectively.
